# Early gestational weight gain and birth weight outcome: a Chinese population-based cohort

**DOI:** 10.1038/s41390-025-04324-2

**Published:** 2025-08-19

**Authors:** Shuang Zhang, Lingyan Feng, Weiqin Li, Nan Li, Leishen Wang, Jia Guo, Jinnan Liu, Junhong Leng, Peng Wang, Huikun Liu

**Affiliations:** Tianjin Women and Children’s Health Center, Tianjin, China

## Abstract

**Background:**

Early gestational weight gain (E-GWG) plays a crucial role in fetal development. Its timing and sex-specific impacts have not been thoroughly investigated, especially in Asian populations.

**Methods:**

In this retrospective cohort study, 66,291 mother-infant pairs from the Tianjin Women and Children’s Health Care System were analyzed. Linear and logistic regression models were applied to examine the association between E-GWG and birth weight outcomes across BMI groups. We also analyzed the effects of E-GWG on the birth weight of infants born to pregnant women carrying male and female fetuses, respectively.

**Results:**

E-GWG had a stronger impact on birth weight z-scores than late GWG (*β* 0.038 vs. 0.016, *P* < 0.001). In each BMI subgroup, E-GWG was positively correlated with the risk of LGA and negatively correlated with the risk of SGA (all *P* < 0.001). The lower the prepregnancy BMI, the more sensitive the birth weight outcomes were to the variation in E-GWG and full-term GWG. The equivalent increase of E-GWG has different effects on the birth weight of male or female newborns. A female newborn has a higher risk of LGA at birth than a male in the underweight, overweight, and obesity group (*P* < 0.05). Girls’ mothers with low pre-pregnancy weight combined with E-GWG less than 10.7 kg had a particularly high risk of SGA. Male newborns have a higher risk of SGA than females with the same insufficient E-GWG in the normal weight and overweight groups. When the E-GWG of mothers with obesity was less than 3.0 kg, the SGA risk was increased for both male (OR 1.764, 95% CI 1.003-3.104) and female newborns (OR 2.540, 95% CI 1.364-4.729) compared with E-GWG more than 8.0 kg.

**Conclusions:**

Insufficient E-GWG of women with underweight highlights the risk of female SGA. Pregnant women with excessive E-GWG should be aware of the risk of LGA, especially those carrying female fetuses.

**Impact:**

- Early GWG showed stronger impacts on birth weight outcomes (LGA/SGA) than late GWG, with BMI/sex effects.
- Female fetuses showed higher LGA odds with excessive E-GWG in moms with overweight/obesity; insufficient E-GWG in moms with underweight strongly correlated with female-specific SGA vulnerability.
- Findings warrant BMI/sex-tailored GWG guidelines to optimize Asian perinatal health and reduce long-term metabolic risks.

## Introduction

The Developmental Origins of Health and Disease framework identifies gestational weight gain (GWG) as a key modulator of fetal programming, with significant impacts on perinatal and long-term health.^1–5^ The Institute of Medicine (IOM) guidelines emphasize GWG monitoring,^3^ but their relevance to Asian populations is unclear.^6–8^ Asian women often have lower prepregnancy body mass index (BMI), unique adiposity distribution, and increased metabolic sensitivity at lower BMI levels.^9–11^ Early gestational weight gain (E-GWG) represents weight gain in the first and second trimesters of pregnancy and is critical for placental vascularization and fetal organogenesis.^12–15^ However, whether E-GWG influences birth outcomes differently by fetal sex and maternal BMI (underweight, normal weight, overweight, obesity) remains underexplored. This study aims to compare the associations of E-GWG versus late GWG (L-GWG) with birth weight z-scores in a large Asian cohort. We also analyze sex-specific effects of the risk of large for gestational age (LGA), and small for gestational age (SGA) related to E-GWG across BMI categories. We hypothesize that E-GWG has a stronger predictive value for birth weight abnormalities (LGA and SGA) than late GWG, with effects modulated by fetal sex and maternal prepregnancy BMI. Clarifying these relationships may refine GWG guidelines to account for fetal sex and maternal BMI, enhancing personalized antenatal care for Asian populations.

## Methods

### Population and data collection

Based on the maternal and infant cohort established by the Tianjin Women and Children’s Health Care System, which covered all communities in Tianjin, China,^16^ this study retrospectively collected the electronic medical records of pregnant women who underwent prenatal care in Tianjin between January and December 2015. We longitudinally tracked all antenatal information from the first prenatal examination to the conclusion of pregnancy, including maternal age, ethnicity, gravidity, parity, date of the last menstrual period, chronic disease history, family medical history, routine examination results (such as height, weight, blood pressure, hematological and urinary laboratory tests, ultrasound findings), pregnancy complications, and delivery outcomes (including the mode of delivery, gestational age at birth, newborn’ sex, birth weight and length). The study protocol was approved by the Institutional Review Board (IRB) of Tianjin Women and Children’s Health Center. To protect patient confidentiality, all collected data went through thorough anonymization. Since this investigation involved a retrospective analysis of routinely collected clinical data, the need for individual informed consent was considered unnecessary. The IRB of Tianjin Women and Children’s Health Center waived the requirement for informed consent. The research was conducted in strict compliance with the ethical principles of the Declaration of Helsinki.

### Inclusion and exclusion criteria

The inclusion criteria were as follows: (a) aged 18–45 years; (b) singleton pregnancy; (c) full-term delivery (gestational age at birth ≥37 weeks and <42 weeks); (d) information on prenatal care and delivery is entirely recorded. The exclusion criteria were as follows: (a) suffering from diabetes or hypertension before this pregnancy; (b) severe anemia, digestive diseases, or tumors; (c) untreated or uncontrolled thyroid disease.

### Measurement

These three hospital-measured weights of pregnant women must be recorded: (a) weight measured at the initial prenatal care (<14 weeks), defined as baseline weight^17,18^; (b) weight measured in 24–28 weeks, defined as weight at the end of the second trimester; (c) weight measured within one week before delivery, defined as delivery weight. Full-term GWG (T-GWG) was defined as the difference between delivery and baseline weights. E-GWG was defined as the difference between weight at the end of the second trimester and baseline weight. L-GWG was defined as the difference between delivery weight and weight at the end of the second trimester. The gestational age at the time of weight measurement was correctly recorded and included in the analysis.

The prepregnancy BMI was calculated based on the baseline weight (kg) divided by the square of the height (m). According to Chinese BMI standards, the mother-infant pairs were divided into four groups^19^: underweight group (prepregnancy BMI < 18.5 kg/m^2^), normal weight group (prepregnancy BMI 18.5–23.9 kg/m^2^), overweight group (prepregnancy BMI 24.0–27.9 kg/m^2^), and obesity group (prepregnancy BMI ≥ 28.0 kg/m^2^). The recommended range of T-GWG in the IOM guidelines was 12.5–18.0 kg, 11.5–16.0 kg, 7.0–11.5 kg, and 5.0–9.0 kg for the underweight, normal weight, overweight, and obese groups.^3^ GWG within the recommended range was defined as appropriate. GWG below the lower limit of the range was defined as insufficient. Excessive GWG was defined as weight gain exceeding the upper limit of the range.

### Observed outcomes of the cohort

The outcomes of this study were the birth weight z-score and weight category at birth (LGA and SGA), which were adjusted for gestational age and fetal sex. Birth weight z-scores were computed utilizing the mean and standard deviation of birth weight derived from our previous population-based study.^20^ Neonatal weight categories were established according to the 2015 Chinese birth weight standards.^21^ Appropriate for gestational age (AGA) was defined as birth weight between the 10 and 90th percentiles, while LGA and SGA were designated as birth weights exceeding the 90th percentile and falling below the 10th percentile, respectively.

### Statistical Analysis

IBM SPSS Statistics for Windows (Version 26.0, Armonk, NY: IBM Corp) and R statistical software (R version 4.2.2, obtained from the Comprehensive R Archive Network) were employed for data analysis and figure generation. Continuous variables following a normal distribution were presented as the mean (standard deviation, SD) and compared using the independent sample *t*-test and one-way analysis of variance (ANOVA). Categorical variables were expressed as counts (percentages) and compared by the *Chi-square* test. GWG was a continuous variable with a non-normal distribution, so it was described by the median (25th percentile, 75th percentile) and analyzed in the form of a continuous variable and a quartile categorical variable, respectively. The influence of GWG on the offspring’s birth weight z-score was evaluated through linear regression analysis. To explore the potential nonlinear relationship between GWG and LGA or SGA, restricted cubic splines (RCS) were plotted. A *P*-nonlinear <0.05 was considered indicative of a non-linear relationship. We showed the estimated ORs and 95%CIs from binary logistic regression models for the associations between E-GWG and the risk of LGA and SGA.

In the multivariate analysis, confounding factors were adjusted for maternal age (in years), ethnicity (Han/others), multiparity (yes/no), education >12 years (yes/no), smoking (yes/no), gestational diabetes mellitus (yes/no), hypertensive disorder of pregnancy (yes/no), and gestational age as weight measurement (in weeks). Due to the intrauterine programming theory of fetal sex specificity, we also analyzed the effects of E-GWG on the birth weight of male and female newborns, respectively. Cases with the z-scores exceeding three SDs were identified as outliers. These data will be verified and collected through system records or telephone inquiries. A two-tailed *P*-value < 0.05 was regarded as statistically significant.

## Results

### Characteristics of pregnant women and their offspring

**Fig. 1:**
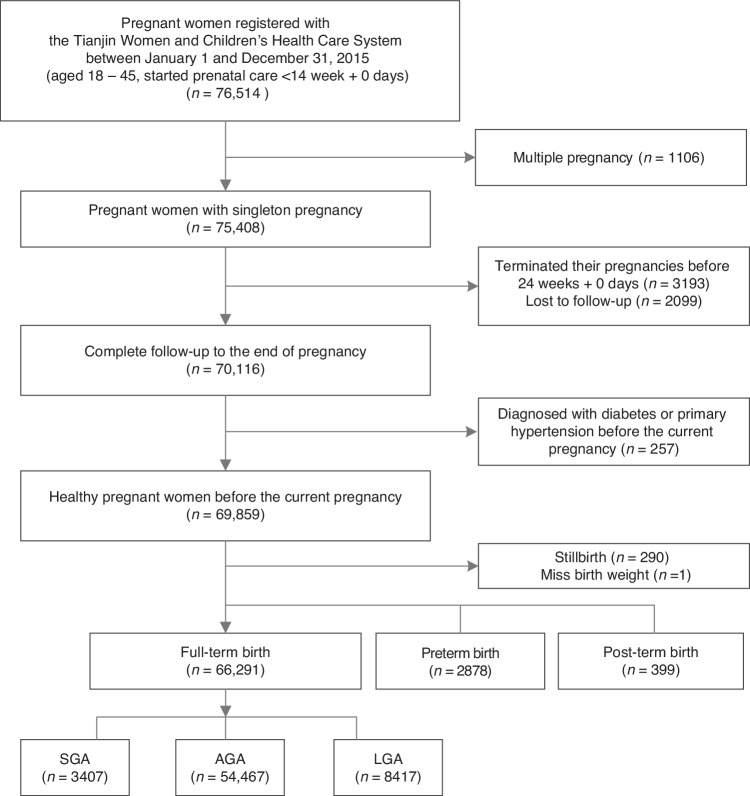
Study Flow Chart. SGA small for gestational age, AGA appropriate for gestational age, LGA large for gestational age.

A total of 66,291 pregnant women who met the inclusion and exclusion criteria were enrolled in the study (Fig. 1). The mean age of these women was 28.14 (4.12) years, with a prepregnancy BMI of 22.62 (3.77) kg/m². Among them, 6821 women (10.3%) were in the underweight group, 39,792 women (60.0%) were in the normal weight group, 13,751 women (20.7%) were in the overweight group, and 5927 women (8.9%) were in the obesity group. The mean gestational age at the initial prenatal examination was 10.80 (1.62) weeks, at the end of the second trimester for weight measurement was 25.96 (0.94) weeks, and at delivery was 39.54 (1.05) weeks. There were 3407 (5.1%) SGA, 54,467 (82.2%) AGA, and 8417 (12.7%) LGA at birth. According to the IOM guidelines, the study population was stratified into three categories: 24,396 women (36.8%) showed appropriate GWG, 13,138 women (19.8%) exhibited insufficient GWG, and 28,757 women (43.4%) exhibited excessive GWG.

Table 1 shows that women with appropriate GWG have later gestational ages at delivery. Pregnant women with excessive weight gain have a younger age at pregnancy, a lower proportion of educated > 12 years, multiparity, gestational diabetes mellitus, and cesarean section, but a higher pre-pregnancy BMI, more smokers, and hypertensive disorder of pregnancy (*P* < 0.01). Moreover, both the absolute value and z-score of birth weight demonstrated a gradual increase (*P* < 0.05) with increased GWG. The proportion of LGA in the excessive GWG and SGA in the insufficient GWG was highest among the three categories (*P* < 0.05). There were no significant differences in ethnicity and fetal sex among the three GWG categories (*P* > 0.05).

**Table 1:** Characteristics of pregnant women in different weight gain categories.

Characteristics	Full-term weight gain categories			
	Insufficient	Appropriate	Excessive	*P*-value
*n*	13138	24,396	28,757	
Maternal age, year	28.58 (4.22)	28.23 (4.10)	27.87 (4.07)	<0.001
Ethnic Han	12,572 (95.7%)	23,332 (95.6%)	27,545 (95.7%)	0.701
Education > 12 years	8599 (65.5%)	15,727 (64.5%)	17,316 (60.2%)	<0.001
Multiparity	4139 (31.5%)	7264 (29.8%)	8298 (28.9%)	<0.001
Smoking	32 (0.2%)	78 (0.3%)	154 (0.5%)	<0.001
Prepregnancy BMI, kg/m^2^	21.91 (3.41)	21.98 (3.55)	23.48 (3.92)	<0.001
Gestational diabetes mellitus	1562 (11.9%)	2402 (9.8%)	2720 (9.5%)	<0.001
Hypertensive disorder of pregnancy	254 (1.9%)	442 (1.8%)	939 (3.3%)	<0.001
Gestational age at delivery, week	39.53 (1.04)	39.55 (1.04)	39.52 (1.05)	0.011
Cesarean delivery	7104 (54.1%)	12,711 (52.1%)	12,555 (43.7%)	<0.001
Infant sex (male)	6716 (51.1%)	12,525 (51.3%)	14,778 (51.4%)	0.873
Birth weight, g	3292.59 (398.81)	3375.61 (405.02)	3505.40 (428.69)	<0.001
Birth weight *z*-score	−0.261 (0.901)	−0.673 (0.920)	0.253 (0.976)	<0.001
Weight category by sex and gestational age at birth				<0.001
SGA	1121 (8.5%)	1420 (5.8%)	866 (3.0%)	
AGA	11,134 (84.8%)	20,595 (84.4%)	22,738 (79.1%)	
LGA	883 (6.7%)	2381 (9.8%)	5153 (17.9%)	

*BMI* body mass index, *SGA* small for gestational age, *AGA* appropriate for gestational age, *LGA* large for gestational age.

### Timing of GWG

**Fig. 2:**
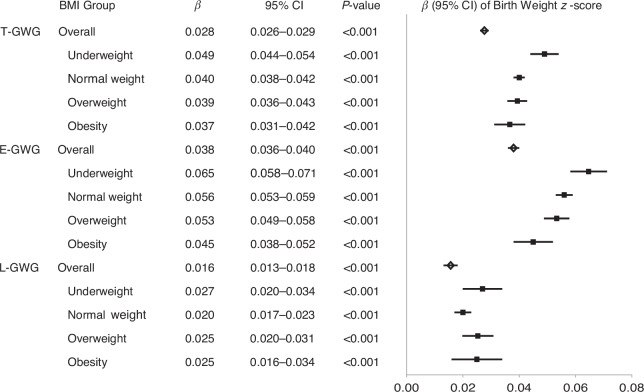
Forest Plot of the Linear Regression between Gestational Weight Gain and Offspring Birth Weight Z-score. Notes: Diamond shape (overall women) and square (BMI group) represent the point estimate; the bars are the 95% confidence intervals. T-GWG full-term weight gain, E-GWG weight gain before the end of the second trimester, L-GWG weight gain after the end of the second trimester, BMI body mass index, CI confidence interval.

To elucidate the relationship between GWG and offspring birth weight, we established linear regression equations. The birth weight z-score was defined as the outcome variable, and T-GWG, E-GWG, and L-GWG as independent variables, respectively. The results demonstrated that T-GWG, E-GWG, and L-GWG all exerted significant influences on the offspring birth weight z-score (all *P* < 0.001) (Fig. 2). Notably, a more pronounced effect was observed for E-GWG (*β* 0.038, 95%CI 0.036–0.040) compared to L-GWG (*β* 0.016, 95%CI 0.013–0.018). This indicates that the weight gain before the end of the second trimester has a powerful impact on the offspring’s birth weight z-score. Furthermore, our analysis revealed the sensitivity of the birth weight z-score to GWG changes across BMI groups. The lower the prepregnancy BMI, the more sensitive the birth weight z-score was to the variation in E-GWG and T-GWG.

### Relationships across BMI groups

E-GWG had the most pronounced effect on birth weight and held clinical value for early assessment and intervention. We further investigated the correlation between E-GWG (a continuous variable) and LGA or SGA. RCS curves explored potential nonlinear correlations between E-GWG and LGA (Supplemental Figure S1) or SGA (Supplemental Figure S2) across different BMI groups. In any BMI subgroup, E-GWG was linearly correlated with the risk of LGA and SGA (all *P* < 0.001, *P*-nonlinear > 0.05). Furthermore, we designed a logistic regression analysis. We took LGA and SGA as the dependent variables (*Y* = 1 for “yes”; *Y *= 0 for “no”), and E-GWG (a continuous variable) as the independent variable. Table 2 indicated that E-GWG had significant impacts on the risks of LGA and SGA in each BMI group (all *P* < 0.001). Both univariate and multivariate analyses were consistent.

**Table 2:** Odd ratios of E-GWG for the risk of LGA and SGA.

Outcome	BMI group	OR (95% CI)	*P*-value	OR (95% CI)	*P*-value
		Model 1		Model 2	
LGA	Under weight	1.162 (1.125–1.201)	<0.001	1.164 (1.126–1.202)	<0.001
	Normal weight	1.129 (1.118–1.140)	<0.001	1.133 (1.122–1.144)	<0.001
	Overweight	1.110 (1.096–1.123)	<0.001	1.116 (1.102–1.129)	<0.001
	Obesity	1.064 (1.048–1.080)	<0.001	1.070 (1.053–1.086)	<0.001
SGA	Under weight	0.847 (0.823–0.872)	<0.001	0.845 (0.821–0.870)	<0.001
	Normal weight	0.873 (0.861–0.885)	<0.001	0.870 (0.858–0.882)	<0.001
	Overweight	0.894 (0.872–0.916)	<0.001	0.888 (0.866–0.910)	<0.001
	Obesity	0.923 (0.892–0.955)	<0.001	0.923 (0.892–0.955)	<0.001

**Note**: Model 1: Univariate regression analysis, with E-GWG (weight gain before the end of the second trimester) as the dependent variable. Model 2: Multivariable regression analysis, adjusting for maternal age (years), ethnicity (Han/others), multiparity (yes/no), education >12 years (yes/no), smoking (yes/no), gestational diabetes mellitus (yes/no), hypertensive disorders of pregnancy (yes/no), and gestational age as weight measured (weeks).

*E-GWG* early gestational weight gain, *LGA* large for gestational age, *SGA* small for gestational age, *BMI* body mass index, *OR* odd ratios, *CI* confidence interval.

### Sexual dimorphism in the risk of LGA and SGA

Table 3 presents results from multivariate logistic regression of E-GWG in women carrying male and female fetuses, respectively. As continuous variables (per 1 kg), the E-GWG was positively associated with LGA risk and negatively associated with SGA risk in both boys’ and girls’ mothers across BMI groups. It is consistent with the conclusion shown in Table 2. As quartile categorical variables, E-GWG also showed a linear trend with the LGA and SGA (*P*-trend < 0.001), similar to the analysis of continuous variables, excluding the mother carrying a male fetus in the obesity group (*P*-trend = 0.175).

**Table 3:** Multivariable odd ratios of E-GWG for the risk of LGA and SGA.

Sex of fetus	BMI Group of mother	OR (95%CI) for E-GWG quartiles					Per 1 kg increase	
		Q1	Q2	Q3	Q4	*P*-trend	OR (95%CI)	*P*
Outcome: LGA								
Male	Underweight	1.00 (ref.)	1.016 (0.562–1.838)	1.543 (0.907–2.626)	3.531 (2.191–5.691)	<0.001	1.160 (1.106–1.216)	<0.001
	Normal weight	1.00 (ref.)	1.434 (1.228–1.673)	1.977 (1.709–2.287)	2.982 (2.593–3.430)	<0.001	1.142 (1.127–1.158)	<0.001
	Overweight	1.00 (ref.)	1.058 (0.876–1.278)	1.489 (1.245–1.780)	2.255 (1.902–2.672)	<0.001	1.113 (1.094–1.132)	<0.001
	Obesity	1.00 (ref.)	1.019 (0.787–1.319)	1.275 (0.996−1.633)	1.637 (1.284–2.087)	<0.001	1.058 (1.035–1.081)	<0.001
Female	Underweight	1.00 (ref.)	0.763 (0.419–1.390)	2.044 (1.256–3.327)	3.691 (2.354–5.788)	<0.001	1.165 (1.114–1.219)	<0.001
	Normal weight	1.00 (ref.)	1.213 (1.044–1.409)	1.664 (1.445–1.917)	2.365 (2.063–2.711)	<0.001	1.115 (1.100–1.131)	<0.001
	Overweight	1.00 (ref.)	1.349 (1.113–1.634)	1.803 (1.497–2.171)	2.496 (2.088–2.984)	<0.001	1.107 (1.088–1.126)	<0.001
	Obesity	1.00 (ref.)	1.386 (1.079–1.781)	1.391 (1.080–1.790)	2.220 (1.744–2.826)	<0.001	1.071 (1.048–1.094)	<0.001
Outcome: SGA								
Male	Underweight	3.468 (2.397–5.018)	1.971 (1.322–2.938)	1.495 (0.992–2.254)	1.00 (ref.)	<0.001	0.860 (0.826–0.896)	<0.001
	Normal weight	3.234 (2.663–3.927)	2.200 (1.795–2.695)	1.615 (1.306–1.996)	1.00 (ref.)	<0.001	0.873 (0.856–0.889)	<0.001
	Overweight	3.463 (2.288–5.241)	2.475 (1.606–3.816)	1.873 (1.193–2.940)	1.00 (ref.)	<0.001	0.886 (0.855–0.917)	<0.001
	Obesity	1.764 (1.003–3.104)	1.445 (0.807–2.588)	1.114 (0.607–2.045)	1.00 (ref.)	0.175	0.930 (0.886–0.975)	0.003
Female	Underweight	5.815 (3.781–8.943)	3.632 (2.318–5.690)	3.156 (1.999–4.983)	1.00 (ref.)	<0.001	0.834 (0.801–0.869)	<0.001
	Normal weight	2.901 (2.375–3.542)	1.793 (1.449–2.218)	1.473 (1.183–1.835)	1.00 (ref.)	<0.001	0.874 (0.856–0.892)	<0.001
	Overweight	2.540 (1.739–3.710)	1.755 (1.172–2.629)	1.210 (0.781–1.874)	1.00 (ref.)	<0.001	0.902 (0.872–0.934)	<0.001
	Obesity	2.540 (1.364–4.729)	1.817 (0.941–3.510)	0.850 (0.390–1.850)	1.00 (ref.)	0.001	0.914 (0.870–0.960)	<0.001

**Note:** Median (25th percentile, 75th percentile) of E-GWG was 8.6 (6.8, 10.7) kg, 8.0 (6.0, 10.2) kg, 7.1 (4.9, 9.4) kg, and 5.5 (3.0, 8.0) kg in the underweight (<18.5 kg/m^2^), normal weight (18.5–23.9 kg/m^2^), overweight (24.0–27.9 kg/m^2^), and obesity (≥28.0 kg/m^2^) group. Q1: 0–25th percentile, Q2: 25–50th percentile, Q3: 50–75th percentile, Q4: 75–100th percentile. Adjusted for maternal age (years), ethnicity (Han/others), multiparity (yes/no), education >12 years (yes/no), smoking (yes/no), gestational diabetes mellitus (yes/no), hypertensive disorders of pregnancy (yes/no), and gestational age as weight measured (weeks).

*E-GWG* weight gain before the end of the second trimester, *LGA* large for gestational age, *SGA* small for gestational age, *BMI* body mass index.

Furthermore, Table 3 showed that for male newborns of mothers with underweight, the risk of LGA was 3.5 times higher with E-GWG > 10.7 kg than with E-GWG < 6.8 kg (OR 3.531, 95%CI 2.191–5.691, *P* < 0.05). In contrast, when a girl’s mother had an E-GWG of just more than 8.6 kg, the risk of LGA was increased by about 2 or more folds (E-GWG 8.6 kg ~10.7 kg: OR 2.044, 95%CI 1.256–3.327, *P* < 0.05; E-GWG > 10.7 kg: OR 3.691, 95% CI 2.354–5.788, *P* < 0.05). It is noteworthy that a slight increase in E-GWG of girls’ mothers significantly affected LGA risk in the overweight (E-GWG 4.9 kg ~7.1 kg: OR 1.349, 95%CI 1.113–1.634, *P* < 0.05) and obesity group (E-GWG 3.0 kg ~5.5 kg: OR 1.386, 95% CI 1.079–1.781, *P* < 0.05). In contrast, the LGA risk of male newborns will be elevated obviously until more increased E-GWG (E-GWG > 7.1 kg in the overweight group and 8.0 kg in the obesity group, *P* < 0.05).

Girls’ mother with low pre-pregnancy weight combined with E-GWG less than 10.7 kg had a particularly high risk of SGA (E-GWG < 6.8 kg: OR 5.815, 95% CI 3.718–8.943, *P* < 0.05; E-GWG 6.8 kg~8.6 kg: OR 3.632, 95% CI 2.318–5.690, *P* < 0.05; E-GWG 8.6 kg~10.7 kg: OR 3.156, 95% CI 1.999–4.983, *P* < 0.05). Correspondingly, an elevated risk of male SGA was significant when the E-GWG was less than 8.6 kg (E-GWG < 6.8 kg: OR 3.468, 95% CI 2.397–5.018, *P* < 0.05; E-GWG 6.8 kg~8.6 kg: OR 1.971, 95% CI 1.322–2.938, *P* < 0.05). In the normal weight and overweight groups, SGA risk was sensitively impacted by E-GWG. Male newborns have a higher risk of SGA than females (e.g., OR 3.234, 95% CI 2.663–3.927 *v.s*. OR 2.901, 95% CI 2.375–3.542 for E-GWG < 6.0 kg in the normal group) with the same weight gain. In addition, when the E-GWG of mothers with obesity was less than 3.0 kg, the SGA risk was significantly increased for both male (OR 1.764, 95% CI 1.003–3.104, *P* < 0.05) and female newborns (OR 2.540, 95% CI 1.364–4.729, *P* < 0.05).

## Discussion

This study suggested that early weight gain assessment during pregnancy can effectively identify LGA and SGA risks. Specifically, mothers with insufficient E-GWG in the underweight group and excessive E-GWG in overweight and obesity groups face heightened sex-specific risks (female fetuses for SGA/LGA), supporting BMI- and sex-tailored GWG guidelines to optimize perinatal outcomes.

In this study, E-GWG (*β* 0.038, 95%CI 0.036–0.040) was significantly more strongly associated with birth weight z-scores than L-GWG (*β* 0.016, 95%CI 0.013–0.018) (Fig. 2), which is consistent with previous epidemiological evidence.^22^ The relationship remained robust after adjustment for potential confounders, including maternal age, prepregnancy BMI, parity, and socioeconomic status (Table 2). This is mainly because the early period coincides with the critical stage of fetal cell proliferation, the rapid development phase of placental nutrient transport capacity, and the sensitive window of maternal metabolism.^23,24^ During this time, maternal nutrient supply directly determines the cellular foundation and growth trajectory of fetal organ development.^25^ In contrast, fetal growth in the third trimester primarily follows a pre-programmed volumetric expansion trajectory established in earlier stages, constrained by physiological limits such as maternal insulin resistance and saturation of placental transport capacity. So L-GWG is more related to maternal health (such as gestational diabetes) and has a limited impact on fetal weight.^26^

This study also suggests that the sensitivity of birth weight to E-GWG varied across maternal BMI categories. The lower the pre-pregnancy BMI, the greater the effect (Fig. 2, Table 2). In the underweight group, GWG more directly impacts neonatal birth weight due to limited maternal reserves, efficient placental nutrient transport during the fetal growth critical period, and a higher proportion of weight allocated to functional tissues (e.g., lean mass, placental development). However, women with overweight and obesity show reduced fetal responsiveness to weight gain due to excessive reserves, metabolic disorders, decreased placental efficiency, and deviation in weight gain synthesis. This difference is essentially the adaptive result of the balance of nutritional supply and demand between the mother and the fetus under different BMI states. It also suggests that in clinical practice, individualized management of weight gain during pregnancy based on BMI is needed to optimize pregnancy outcomes.

The impact of maternal GWG at different stages of pregnancy may vary between male and female infants.^27–30^ This study also found that the association between E-GWG and birth weight shows fetal sex dimorphism. Previous studies have suggested that male fetuses grow rapidly in the uterus, are more sensitive to insufficient nutrition and oxygen supply, have limited compensatory capacity of the placenta, and are prone to structural defects (such as reduced nephrons and insufficient cardiomyocytes).^27^ However, female fetuses grow slowly in utero, and the placenta preferentially ensures energy reserves by upregulating glucose transporters (such as GLUT3) and IGF-2 receptors. It may lead to female fetuses being more prone to overgrowth when the mother’s metabolism is abnormal. As the results of this study show (Table 3), female infants in the overweight and obesity groups have a higher risk of LGA. On the other hand, female fetuses may compensate for excessive growth through efficient placental transport under extreme nutritional stress, which shows a significantly increased risk of female LGA at birth in the underweight group.

The risk of SGA in female fetuses was higher in the underweight group (Table 3). When her E-GWG was less than 6.8 kg, the risk of SGA was 5.815 (95%CI 3.781–8.943) folds than that of E-GWG more than 10.7 kg. It may be related to the higher efficiency of placental glucose transport in female fetuses and their greater sensitivity to energy deficiency.^27^ Even if the mother’s nutritional reserves are insufficient, she can still prioritize the intake of glucose and convert it into fat for deposition. Male fetuses have a higher risk of SGA in normal/overweight pregnant women (Table 3). This may be related to the fact that male fetuses rely on amino acid-driven lean body mass gain and are more sensitive to protein deficiency.^27^

The mechanisms underlying the relationship between maternal obesity and either low or high neonatal birth weight are not yet fully understood. Besides genetic factors, placental function, maternal nutritional status, and the transfer of nutrients from the mother to the fetus are considered the main determinants of fetal growth.^31–33^ Placental abnormalities and other factors (such as maternal diseases) slow fetal growth by impairing placental function and are associated with the occurrence of low birth weight (LBW) and fetal growth restriction (FGR).^34,35^ Future research needs to further investigate the mechanisms behind these sex-specific influences.

## Conclusion

Weight management during the first and second trimesters of pregnancy should be individualized based on pre-pregnancy BMI and the sex of the fetus. The clinical significance of this study lies in that, through targeted intervention, it may reduce the risks of LGA or SGA, optimize maternal and infant outcomes, and demonstrate the key regulatory role of early precise management in fetal development programming.

## Supplementary information

Supplementary Information

## Data Availability

The datasets generated during and/or analyzed during the current study are not publicly available because the data in this study are public health data and are protected by government security laws and regulations, but are available from the corresponding author on reasonable request.
