# Supplementary material for: Early gestational weight gain and birth weight outcome: a Chinese population-based cohort
Source: Pediatr Res. 2025 Aug 19;99(3):929–35. doi: 10.1038/s41390-025-04324-2 (PMC13021518; doi:10.1038/s41390-025-04324-2)
Supplement: Supplementary file 1 — Supplementary Information [file 41390_2025_4324_MOESM1_ESM.pdf]

Supplemental file

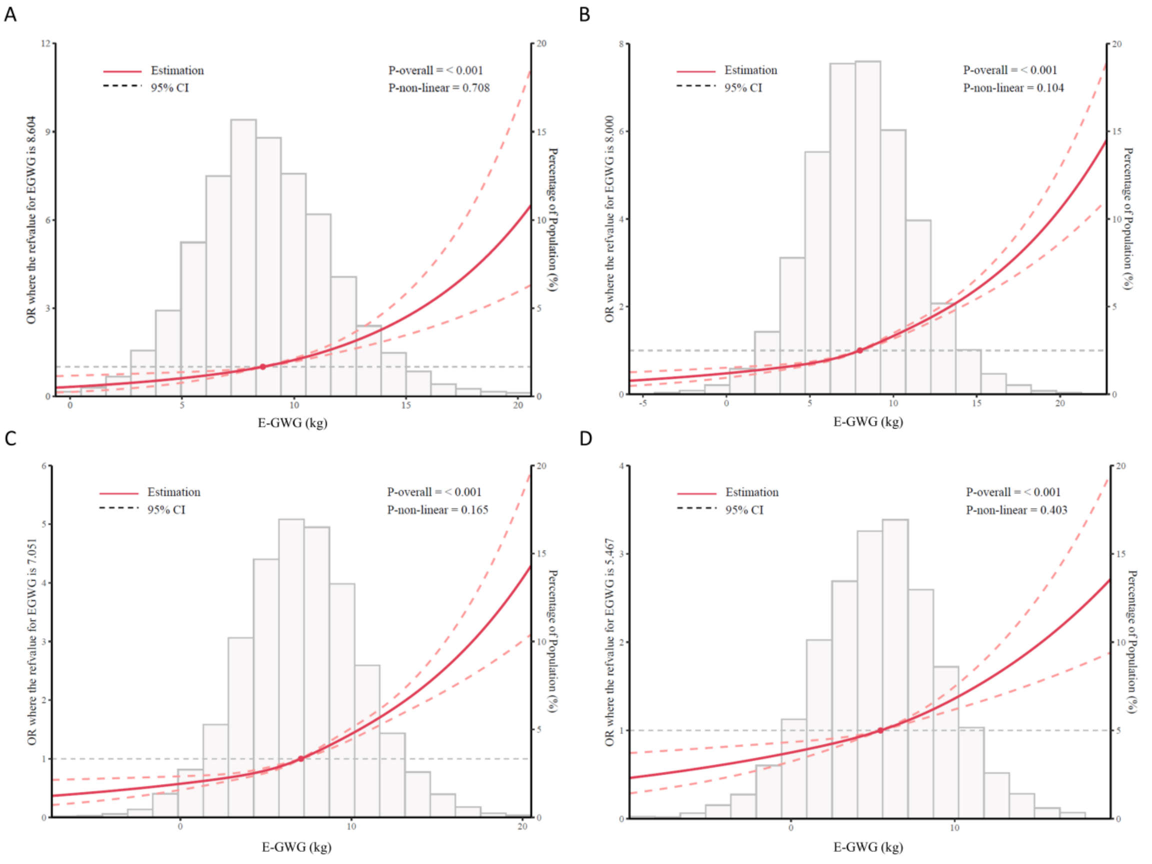

Supplemental Figure S1. Restricted cubic spline for the associations between early gestational weight gain and the risk of large for gestational age infant.

A. underweight group, B. normal weight group, C. overweight group, D. obesity group. Prepregnancy BMI groups were divided following the Chinese standard: underweight, <18.5 kg/m^2; normal weight, 18.5–23.9 kg/m^2; overweight, 24.0–27.9 kg/m^2; obesity, ≥ 28.0 kg/m^2. The curves represent OR (solid lines) and 95% CI (long dashed lines) for the effect of GWG at the end of the second trimester (E-GWG) on LGA. The model was adjusted for maternal age (years), ethnicity (Han/others), multiparity (yes/no), education >12 years (yes/no), smoking (yes/no), gestational diabetes mellitus (yes/no), hypertensive disorders of pregnancy (yes/no), and gestational age as weight measured (weeks). The reference values were set at the 50th percentiles (OR=1). The knots in the default positions were placed at the 5th, 35th, 65th, and 95th percentiles of the E-GWG. The histograms represent the distribution of E-GWG in the cohort.

Abbreviations: E-GWG, early gestational weight gain; BMI, body mass index; OR, odd ratios; CI, confidence interval; LGA, large for gestational age.

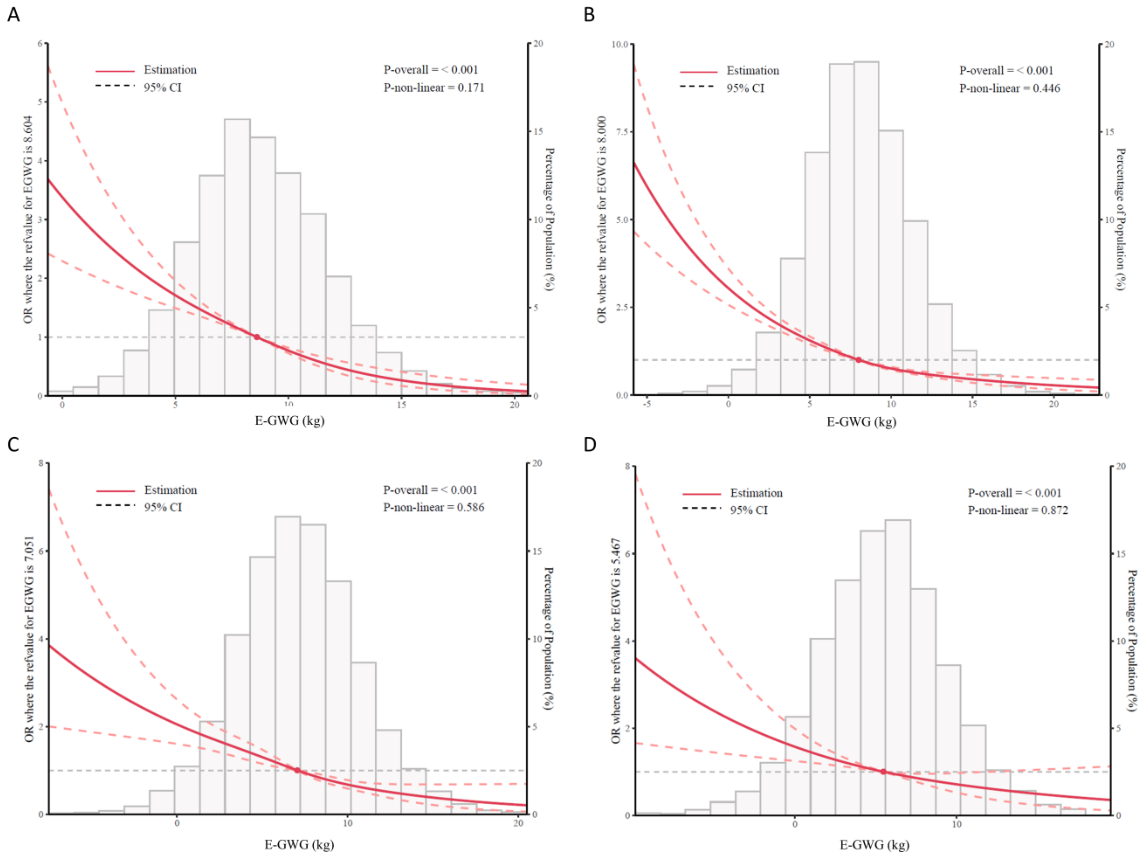

Supplemental Figure S2. Restricted cubic spline for the associations between early gestational weight gain and the risk of small for gestational age infant.

A. underweight group, B. normal weight group, C. overweight group, D. obesity group. Prepregnancy BMI groups were divided following the Chinese standard: underweight, <18.5 kg/m^2; normal weight, 18.5–23.9 kg/m^2; overweight, 24.0–27.9 kg/m^2; obesity, ≥ 28.0 kg/m^2. The curves represent OR (solid lines) and 95% CI (long dashed lines) for the effect of GWG at the end of the second trimester (E-GWG) on SGA. The model was adjusted for maternal age (years), ethnicity (Han/others), multiparity (yes/no), education >12 years (yes/no), smoking (yes/no), gestational diabetes mellitus (yes/no), hypertensive disorders of pregnancy (yes/no), and gestational age as weight measured (weeks). The reference values were set at the 50th percentiles (OR=1). The knots in the default positions were placed at the 5th, 35th, 65th, and 95th percentiles of the E-GWG. The histograms represent the distribution of E-GWG in the cohort.

Abbreviations: E-GWG, early gestational weight gain; BMI, body mass index; OR, odd ratios; CI, confidence interval; SGA, small for gestational age.
